# Actigraphic estimates of sleep duration in those reporting sleeping less than 7 h

**DOI:** 10.1007/s44470-026-00072-0

**Published:** 2026-05-22

**Authors:** Kelly Glazer Baron, Joshua Landvatter, Alena Wilson, Jennifer Duffecy, Adam Bress, Molly B. Conroy, Sara E. Simonsen, Chelsea Allen, Tom Greene

**Affiliations:** 1 Department of Family Medicine and Public Health, University of Utah, Salt Lake City, UT USA; 2 Department of Psychiatry, University of Illinois at Chicago, Chicago, IL USA; 3 Department of Population Health Sciences, University of Utah, Salt Lake City, UT USA; 4 Department of Internal Medicine, University of Utah, Salt Lake City, UT USA; 5 College of Nursing, University of Utah, Salt Lake City, UT USA

## Abstract

**Objectives:**

Sleep duration < 7 h increases risk for chronic disease, which makes identifying short sleep duration critical to public health. The goal of this study is to evaluate objective short sleep duration among individuals who self-report insufficient sleep to test and evaluate predictors of the subjective–objective sleep duration difference.

**Methods:**

This study presents baseline data from a sleep extension study involving adults aged 18–65, fluency in English, and self-reported sleep duration ≤ 7 h and elevated blood pressure. Objective sleep duration was measured with actigraphy, and subjective–objective sleep difference was calculated as the difference between self-reported habitual sleep duration and actigraphically measured sleep duration. Data were analyzed using regression models, Bland–Altman plots and exploratory spline-based logistic regression models.

**Results:**

Among 195 adults (age *m* = 42 ± 11 years), 54% had objective sleep duration < 7 h, and on average participants underestimated their sleep by 29 m. Under-reporting of self-reported sleep compared to actigraphy was associated with poorer objective sleep, including longer sleep onset latency (*p* < .001) and higher wake after sleep onset (*p* < .001), but also higher sleep efficiency (*p* < .001). In addition, perceived stress (*p* < .01) and self-reported sleep disturbance (*p* < .01) were associated with underestimation. A Bland–Altman analysis showed larger negative differences at longer objective sleep durations, consistent with both sleep perception patterns and statistical regression effects. Exploratory spline-based logistic modeling indicated a U-shaped relation with the lowest predicted probability of a large subjective–objective difference occurring at approximately 6.3 h of objective sleep duration.

**Conclusion:**

These findings highlight the importance of objective assessments to determine short sleep duration. Poorer subjective and objective sleep and higher stress may intensify perceptions of inadequate sleep, contributing to under-reporting.

**Brief Summary:**

Sleep duration < 7 h increases risk for chronic disease, which makes identifying short sleep duration critical to public health. The goal of this study is to evaluate objective short sleep duration among individuals who self-report insufficient sleep and test and evaluate predictors of the subjective–objective sleep duration difference. Results demonstrate that half of individuals with self-reported sleep duration < 7 h did not have objective short sleep duration; most participants tended to underestimate their sleep duration, in particular those with poorer objective sleep, sleep disturbance, and higher stress. This difference between self-reported and objective sleep highlights the complexity of identifying individuals and populations with objectively short sleep duration for research and public health interventions.

## Introduction

Approximately one-third of adults in the USA sleep less than the recommended 7 h per night [1]. Insufficient sleep is consequential to public health due to increased risk for obesity, cardiovascular disease, decreased quality of life, accidents and workplace injuries [2]. The determinants of insufficient sleep are multifactorial and include sleep disorders, work and commute times as well as leisure activities [3, 4]. Given the public health importance of encouraging adequate sleep duration, it is important to identify and motivate behavior change among those who need to increase sleep duration.

According to the Health Behavior Model [5], individuals must perceive themselves at risk and be aware of the consequences of their behavior to motivate action. This model was applied to sleep by Knowlden et al., [6] who found that health behavior model features explained 34% of the variance in college students’ self-report sleep duration. However, sleep duration estimates vary based on how they are measured, with studies reporting different health-related correlates of self-report versus objective assessments [7, 8]. Although objective estimates of sleep such as actigraphy are moderately correlated with self-report, how self-reported sleep is collected, even for a single estimate of self-reported sleep, can impact the estimate. For example, a single self-reported sleep duration item has a lower correlation compared with more precise reports of self-reported sleep (e.g., bedtime, wake time, sleep latency, and wake after sleep onset), as is used in the consensus sleep diary [9]. It is also important to consider that actigraphy is an estimation of sleep via motion-based activity counts and tends to overestimate sleep compared to polysomnography.

Most epidemiologic studies have shown that individual’s self-reported sleep duration is longer than objective measurement using actigraphy. In the CARDIA Study, participants overall overestimated their sleep on self-report by nearly an hour compared to their actigraphy recording, using either a single item or a diary-based estimate to measure self-report estimates [7] In the Osteoporotic Fractures in men’s and women’s studies, nearly half of the participants self-reported daily sleep duration between 6 and 8 h on a single item when estimating their sleep in the past month, but demonstrated sleep duration < 6 h on actigraphy [10]. Additionally, in the Hispanic Community Health Study/Study of Latinos, participants overestimated their sleep duration by more than 1 h on average compared with actigraphy measurements [11].

Previous studies have evaluated demographic and health characteristics that are associated with a greater subjective–objective sleep duration difference. For example, in the CARDIA study, Black participants, those with obesity, higher daytime sleepiness, and higher sleep efficiency, had less subjective–objective difference [7]. Also, in the Hispanic Community Health Study/Study of Latinos, subjective–objective sleep duration difference was lower among males, younger participants, those with lower sleep efficiency and more sleep variability [11]. Subjective–objective sleep duration difference has been extensively studied in individuals with insomnia, who are generally found to underestimate their sleep duration by self-report; however, this pattern is inconsistent, as individuals with insomnia do not always differ from controls in their tendency to under- or overestimate sleep duration [9]. These studies together suggest that demographics, health characteristics, and sleep quality may influence how sleep is subjectively perceived, but there is not agreement between studies.

Our team is interested in understanding simple measures for identifying individuals with short sleep duration, to identify those who are likely to have objective (actigraphic) sleep duration < 7 h and to inform the development of sleep extension interventions to promote health and well-being [12]. In contrast to epidemiologic studies that are trying to understand bias in self-report compared to objective sleep duration, the goal of this analysis is to better identify who is likely to benefit from interventions designed to improve sleep, a critical step in translating findings from research into clinical and public health application. Given that most studies show that the tendency is for individuals to overestimate their sleep, there may be individuals who are at risk but do not perceive themselves as needing intervention. On the other hand, individuals who perceive their sleep duration as short despite obtaining an adequate amount of sleep may not respond to a sleep extension intervention. Therefore, the goal of this study is to examine subjective–objective sleep duration difference among participants who completed a brief 1–2 item prescreening measure of habitual sleep for a sleep extension trial and later participated in objective screening with actigraphy. A key difference between this study and others is that participants in this study volunteered because they were interested in increasing their sleep duration. Results of this study will inform future sleep extension research and further the understanding of sleep perception among short sleepers.

## Methods

### Participants and procedure

This study is an analysis of the screening/baseline data collected in the Sleep Technology Intervention to Target Cardiometabolic Health (STITCH) study [13]. The STITCH study is a parallel group, randomized controlled study to test the effects of a behavioral sleep extension intervention compared to a health education control group on sleep duration and cardiometabolic health among adults with objective sleep duration less than the recommended optimal levels according to a recent consensus statement (< 7 h per night) with elevated blood pressure. Participants were recruited to complete the screening questionnaire using various recruitment methods including mailed letters based on electronic medical records, phone calls, email communications, social media advertisements, and flyers distributed throughout the community. Recruitment materials advertised that the study was recruiting men and women with blood pressure above 120/80, sleep less than 7 h, and are interested in increasing their sleep duration. Potentially eligible participants completed a brief eligibility survey which included self-reported sleep duration. Those who were eligible for additional screening completed informed consent and then wore actigraphy for 10 days to verify objective sleep duration criteria and collect baseline questionnaires. Eligible participants were aged 18–65 years, self-reported average sleep duration ≤ 7 h per night and time in bed < 8 h (via actigraphy), had smartphone access, and were fluent in English. Although suboptimal sleep duration was defined as < 7 h, several participants with 7 h of sleep were included in screening due to the previous literature suggesting most self-report estimates are longer than objective estimates on actigraphy.

### Measures

Demographic measures included age, sex, race, employment, and relationship status.

Subjective habitual sleep duration was measured at pre-screening with the following single item at the beginning of the study “How many hours of sleep do you usually sleep each night.” We later updated this measure at prescreening to two items. An average self-report sleep duration was created by calculating the number of weekdays and weekends (weekday sleep duration $\times$ 5 + weekend sleep duration $\times$ 2)/7. There was not a statistically significant difference in sleep duration estimates from the 1 item versus the 2 item measure (*n* = 59 with 1 item and *n* = 136 with 2 items); thus, we combined these measures in the analysis for the measure of subjective habitual sleep duration.

Objective sleep duration was estimated using the Actiwatch Spectrum device (Philips Respironics Inc.) worn for at least 7 days and results were analyzed in the Actiware program. Rest intervals were manually scored by blinded research staff using a standardized protocol [14]. Rest periods were identified using event markers recorded by the participant and if not available, the rest interval was scored based on a drop in light and activity. If a participant had off-the-wrist time during the rest period, the night was excluded. Variables used in the analyses included sleep duration, sleep onset latency (SOL), wake after sleep onset (WASO), and sleep efficiency (SE). SE was calculated as the ratio of total sleep time to total time in bed (i.e., total sleep time/total time in bed).

Subjective objective sleep duration difference was calculated as self-reported sleep duration minus objective sleep duration measured by actigraphy. A positive number indicates overestimation of subjective sleep duration compared to objective sleep duration and a negative number indicates underestimation of subjective sleep duration compared to objective sleep duration.

PROMIS sleep disturbance and sleep-related impairment 8-item questionnaires were used to measure sleep quality [15]. These questionnaires have been found to have excellent psychometric properties for quantifying subjective characteristics of adult sleep quality. Scores on this measure are transformed to *t*-scores with a mean of 50 and standard deviation of 10.

Depressive symptoms were measured by the Patient Health Questionnaire eight item (PHQ-8) [16]. The questionnaire is scored between 0 and 24, asking the respondent to report the frequency of experiencing each depressive symptom within the last 2 weeks. Higher scores indicate higher depressive symptoms. Scores on this measure are highly correlated with clinical diagnoses of depression.

The perceived stress scale, 10-item (PSS-10) was utilized as a brief measure of stress ratings [17]. This widely used measure has been used to characterize global stress and previous studies have associated PSS scores with shorter sleep duration in various populations [18, 19]. Items are rated from 0 to 4 and scores range from 0 to 40, with higher scores indicating higher stress.

Insomnia symptoms were measured by the insomnia severity index (ISI) [20]. The ISI is comprised of 7 items scored from 0 to 4 with higher scores indicating that the respondent experiences more severe insomnia and associated symptoms. The ISI has been demonstrated to be valid for quantifying insomnia symptoms in clinical and community samples with reliable internal consistency.

The Epworth Sleepiness Scale (ESS) is a widely used 10-item measure of daytime sleepiness [21, 22]. ESS scores can range from 0 to 24. Respondents are asked to rank their likelihood of dozing from 0 = “would never doze” to 3 = “high chance of dozing” for eight different daytime scenarios.

Sleep apnea severity was assessed for all participants using one night of home sleep apnea testing with the ApneaLink monitor (ResMed Inc. Poway, CA). This device has been validated as a reliable estimate of sleep apnea diagnosis in the home environment [23]. The apnea–hypopnea index (AHI) was calculated as the number of apneas and hypopneas per hour of recording.

### Statistical analyses

All analyses were performed using R statistical software (R Core Team, 2024). Demographics were summarized with means and frequencies. Subjective–objective sleep duration difference was examined descriptively and illustrated using a Bland–Altman plot to evaluate bias and agreement between self-reported and actigraphy-measured sleep duration. Associations between subjective–objective sleep duration difference and actigraphic variables (sleep efficiency, sleep onset latency, and wake after sleep onset) were evaluated using regression models and visualized using cubic spline-based curves.

To formally evaluate predictors of subjective–objective sleep duration difference, linear regression models were used. For each predictor, three models were estimated: (1) a partially adjusted model controlling for age, race, and sex; (2) a fully adjusted objective model including age, race, sex, AHI, SOL, WASO, and SE; and (3) a fully adjusted subjective model including age, race, sex, AHI, depression, insomnia, perceived stress, daytime sleepiness, sleep disturbance, and sleep-related impairment. Regression results are presented in Table 1 (objective predictors) and Table 2 (subjective predictors). Because visual inspection of the sleep efficiency plot suggested potential non-linear patterns, exploratory analyses were conducted. Exploratory spline-based logistic regression models, using natural cubic splines with two degrees of freedom beyond linearity, were used to examine whether objective sleep duration predicted high subjective–objective sleep duration difference (≥ 45 min, the top 1/3rd of the sample; Fig. 4). Results from linear regression models are reported as β coefficients with 95% confidence intervals and *p*-values, with statistical significance defined as *p* < 0.05.

**Table 1:** Regression results: actigraphic measures predicting sleep difference

	Partially adjusted				Fully adjusted			
	*β*	95% CI		*p*	*β*	95% CI		*p*
Sleep onset latency (SOL)	− 0.0131	− 0.0279	0.0017	0.0686	− 0.0493	− 0.0656	− 0.0328	< 0.001***
Wake after sleep onset (WASO)	− 0.0280	− 0.0383	− 0.0177	< 0.001***	− 0.0695	− 0.0814	− 0.0575	< 0.001***
Sleep efficiency (SE)	0.0086	− 0.0191	0.0363	0.537	− 0.2024	− 0.2458	− 0.1589	< 0.001***

*Note.* Partial models are adjusted for age, sex, and race. Full models are adjusted for age, sex, race, and all other actigraphic measures in the table. *p* <.001 (***). Sleep difference is expressed in hours. Coefficients for SOL and WASO represent the change in sleep difference per 10-min increase in the predictor. Coefficients for sleep efficiency represent the change in sleep difference per 5-percentage-point increase in efficiency

**Table 2:** Regression results: self-reported sleep measures predicting sleep difference

	Partially adjusted				Fully adjusted			
	*β*	95% CI		*p*	*β*	95% CI		*p*
Depression Severity (PHQ8)	0.0182	− 0.0280	0.0644	0.399	0.0431	− 0.0032	0.0894	0.0700
Insomnia Severity Index (ISI)	− 0.0497	− 0.0859	− 0.0135	0.0077**	− 0.0351	− 0.0828	0.0126	0.1503
Perceived Stress Scale (PSS)	− 0.0735	− 0.1421	− 0.0049	0.0373*	− 0.0948	− 0.1643	− 0.0253	0.0083**
Epworth Sleepiness Scale (ESS)	0.0136	− 0.0247	0.0519	0.455	0.0248	− 0.0135	0.0631	0.2052
PROMIS sleep disturbance	− 0.0403	− 0.0619	− 0.0187	0.0003***	− 0.0420	− 0.0720	− 0.0120	0.0068**
PROMIS sleep impairment	− 0.0134	− 0.0550	0.0282	0.1099	− 0.0067	− 0.0275	0.0141	0.5282

*Note.* Partial models are adjusted for age, sex, and race. Full models are adjusted for age, sex, race, and all other self-reported measures in the table. *p* <.05 (*), *p* <.01 (**), *p* <.001 (***). Coefficients represent the change in sleep difference (hours) per 1-point increase in the respective self-reported scale

## Results

### Participant characteristics and description of subjective–objective sleep duration difference

**Fig. 1:**
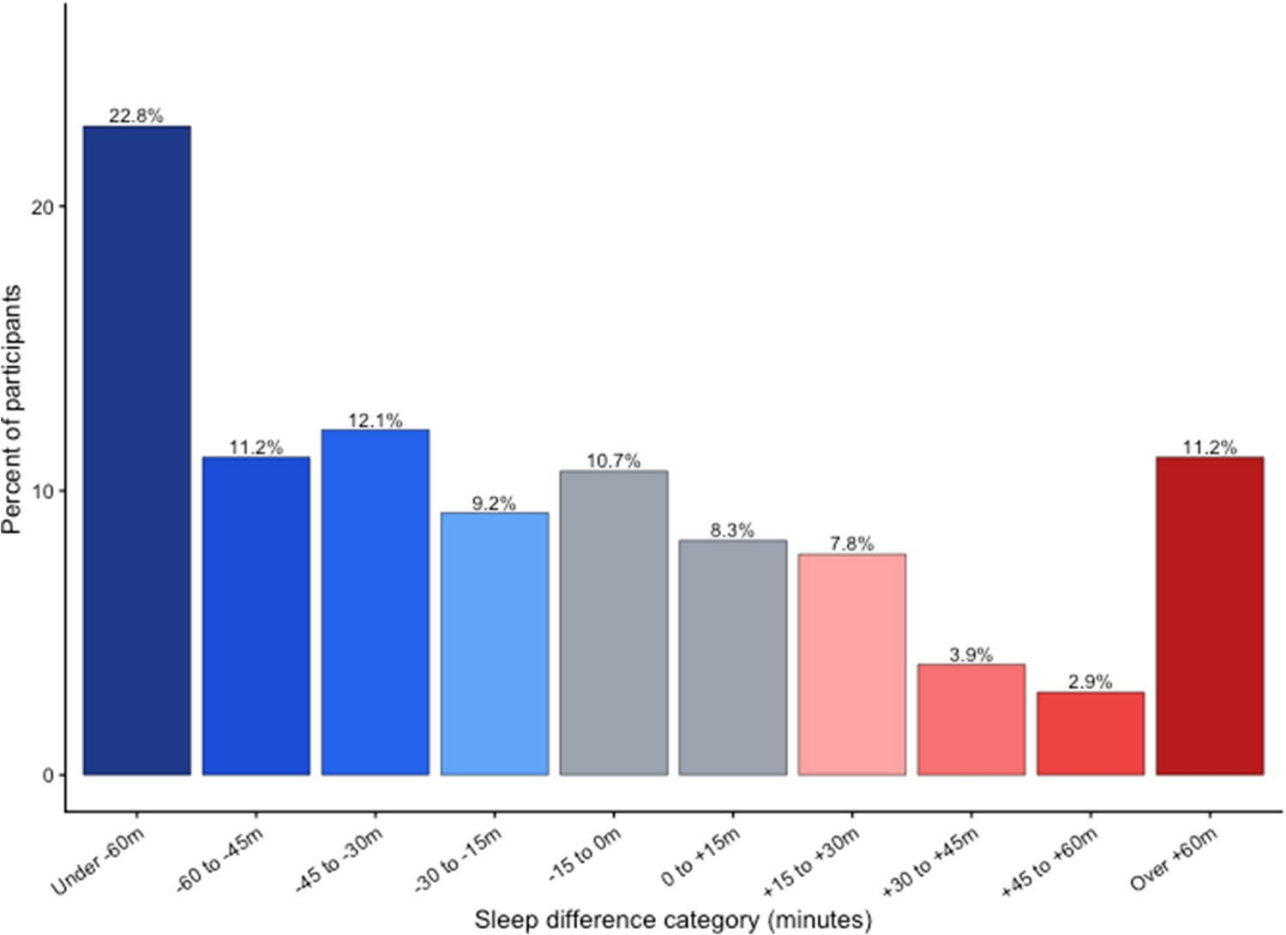
Distributions of differences between prescreening self-reported sleep duration and subsequent actigraphic sleep duration at screening/baseline

**Fig. 2:**
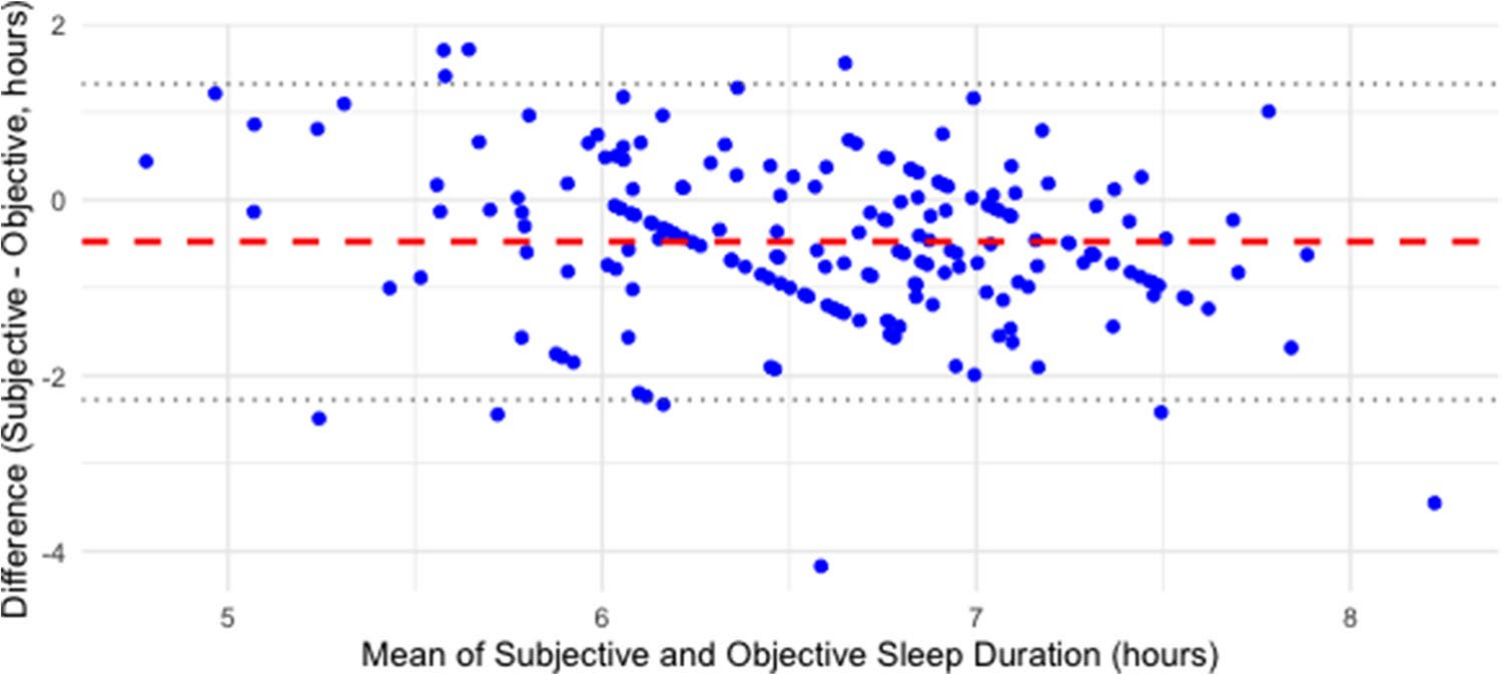
Bland–Altman plot: self-reported sleep duration at prescreening vs. objective sleep duration at the baseline assessment

A total of 212 participants completed actigraphy and 17 participants were excluded from this analysis due to incomplete actigraphy data (< 5 nights of usable sleep data), resulting in 195 participants in this analysis. Participant characteristics are listed in Table 3 (see below). All included participants had self-reported sleep < 7 h. Mean age was 42 years (SD = 11) and 36% were female. Slightly more than half (54.4%) demonstrated sleeping < 7 h on actigraphy. There was a positive correlation between self-reported sleep duration and actigraphy-measured sleep duration (*r* = 0.31, 95% CI [0.18, 0.43], *p* < 0.001). On average, the difference score was − 29 min, indicating that the self-report sleep is about a half hour lower than the objective sleep duration on actigraphy. Figure 1 shows that 24.4% subjectively underestimated their sleep by more than 60 min and smaller proportions underestimated by 45–60 min (11.9%) and 30–45 min (13.0%). Approximately one-fifth of participants reported sleep within 15 min of their objective sleep duration. Overestimation was less common, with fewer participants overestimating by more than 15 min. Overall, underestimation was more prevalent than overestimation in this sample. A Bland–Altman plot comparing subjective and objective sleep duration (Fig. 2) showed a small average bias toward underestimation, consistent with the mean difference of approximately − 29 min.

**Table 3:** Participant characteristics

Variable	Mean ± SD or *N* (%)	Range
Age (years)	42.12 ± 11.1	21–67
Sex (male, %)	124 (63.6%)	—
Race (%)		—
-White	140 (71.8%)	—
-Asian	27 (13.8%)	—
-Black	4 (2.1%)	—
-Other (American Indian/Alaskan Native, Native Hawaiian/Pacific Islander, more than one race, not reported)	24 (12.3%)	—
BMI (kg/m [2])	32.1 ± 18.8	19.0–53.6
Apnea–hypopnea index (AHI)	8.7 ± 11.0	0.2–82.8
Self-report sleep duration, h	6.3 ± 0.7	4.0–7.0
Sleep efficiency, actigraphy, %	87.4 ± 5.1	66.2–96.0
Total sleep time, actigraphy, h	6.8 ± 0.9	4.4–10.0
Wake after sleep onset (WASO), actigraphy, m	30.7 ± 13	9.3–91.1
Sleep onset latency (SOL), actigraphy, m	11.2 ± 15.5	0–117.5
Patient Health Questionnaire-8 (PHQ8)	4.7 ± 3.3	0–13.0
Epworth Sleepiness Scale (ESS)	6.8 ± 3.7	0–18.0
Perceived Stress Scale (PSS)	9.1 ± 2.0	0–15.0
Insomnia Severity Index (ISI)	8.9 ± 3.7	0–14.0
PROMIS sleep disturbance	52.2 ± 5.9	33.5–69.0
PROMIS sleep-related impairment	52.8 ± 8.2	26.2–74.8

#### Associations with actigraphic variables (Table 1)

**Fig. 3:**
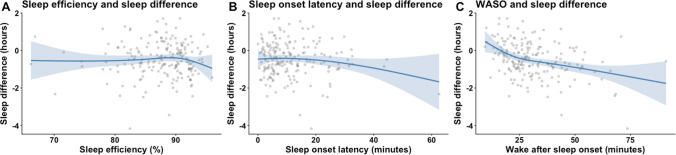
Subjective–objective sleep duration difference across objective sleep metrics

**Fig. 4:**
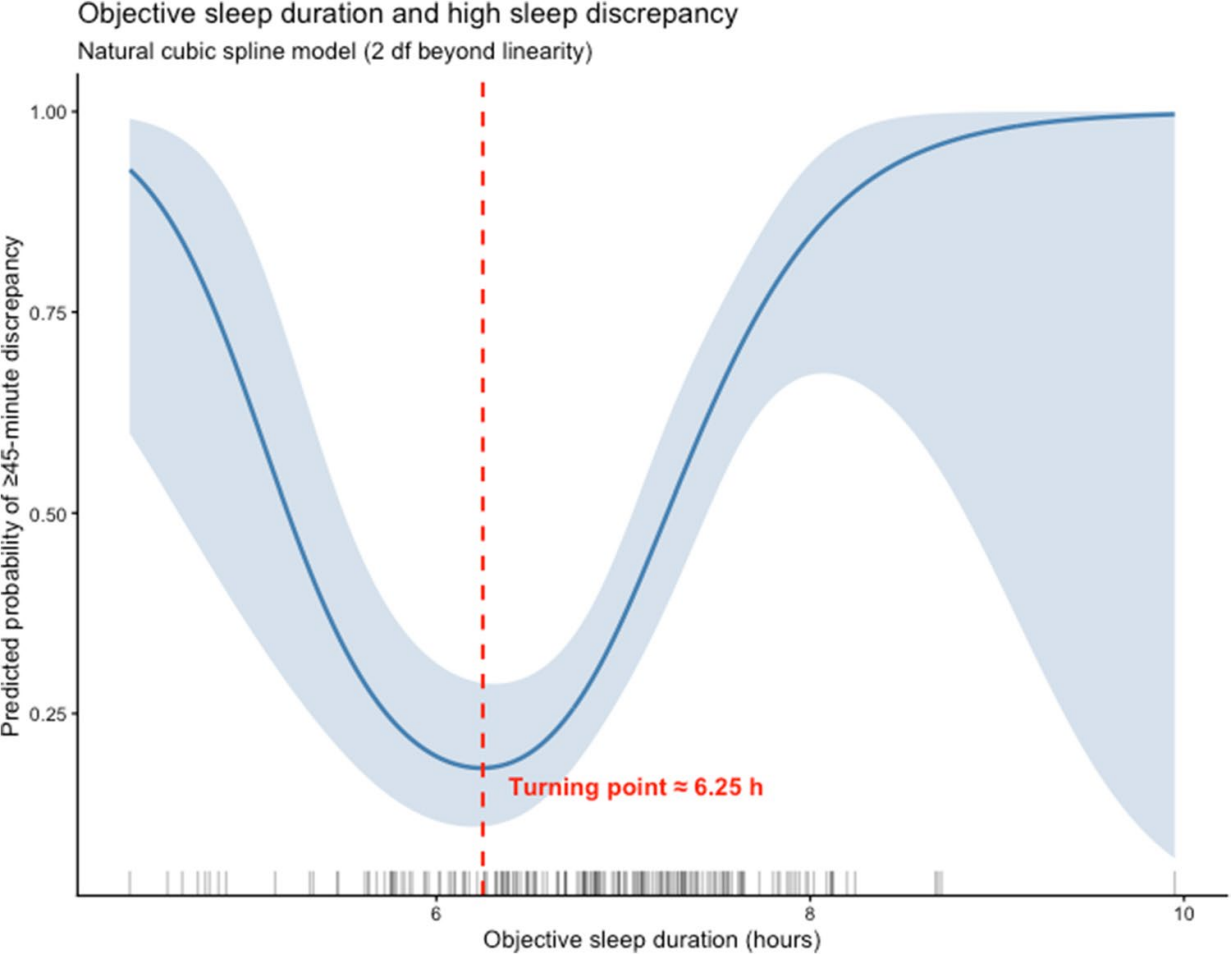
Predicted probability of ≥ 45 min subjective–objective sleep duration difference

Figure 3 illustrates the relationships between subjective–objective sleep duration difference and three actigraphic sleep metrics: sleep efficiency, sleep onset latency and WASO. Participants with poorer actigraphy scores had greater subjective underestimation of sleep duration (e.g., lower self-report compared to objective). More specifically, longer sleep onset latency (SOL, full model *β* = − 0.0493, *p* < 0.001) and higher wake after sleep onset (WASO, partially adjusted model *β* = − 0.0280, *p* < 0.001, full model *β* = − 0.0695, *p* < 0.001) were associated with a greater subjective underestimation of sleep duration. Greater SE was also associated with greater underestimation of sleep duration in the full model, after controlling for demographics, AHI, and other actigraphy variables (*β* = − 0.2024, *p* < 0.001). Visual inspection of the sleep efficiency plot (see Fig. 3) indicates an inflection point around 85% sleep efficiency. Specifically, individuals with lower sleep efficiency (below ~ 85%) appear more likely to overreport their objective sleep duration, while those with higher sleep efficiency (above ~ 85%) tend to underreport their sleep duration. Although the overall regression indicates a negative association between sleep efficiency and subjective–objective sleep duration difference, this suggests a potentially non-linear relationship that may be masked when only considering the aggregate slope. When exploratory spline-based logistic regression modeling of objective sleep duration as a predictor of high subjective–objective sleep duration difference (≥ 45 min, the highest 1/3rd of the sample) was conducted, the resulting curve indicated a U-shaped relationship. 2 The predicted probability of high difference was lowest at approximately 6.3 h of objective sleep duration, with higher probabilities observed at both shorter and longer sleep durations (see Fig. 4). AHI, age, sex, race, or ethnicity were not significantly associated with subjective–objective sleep duration difference in any of the models.

#### Associations with self-reported questionnaires (Table 2)

Higher PROMIS sleep disturbance scores (partially adjusted *β* = − 0.040, *p* < 0.001, full model, *β* = − 0.042, *p* = 0.007) and perceived stress scores were significantly related to greater underestimation of sleep in both the partially adjusted models (*β* = − 0.0734, *p* = 0.037) and full model (*β* = − 0.095, *p* = 0.008). Higher ISI scores were associated with greater underestimation of sleep in the partially adjusted model (*β* = − 0.0497, *p* = 0.008) but not in the full model after adjusting for demographics, AHI, and all other self-report questionnaires (sleep disturbance, depressive symptoms, daytime sleepiness). Demographics (age, sex, race or ethnicity), depressive symptoms on the PHQ, daytime sleepiness on the ESS, and PROMIS sleep-related impairment were not associated with subjective–objective sleep duration difference.

## Discussion

The goal of this study was to examine the difference between participants’ subjective sleep duration reports at prescreening and later screening actigraphy among individuals who self-identified as sleeping 7 h or less and applied to participate in a study of sleep extension among adults with elevated blood pressure. This study found that on average, individuals’ retrospective self-reported sleep duration was about a half hour less than objective recording with actigraphy. However, there was a range of both over and under-reporting. These findings contrast with prior population-based studies which have generally found that subjective sleep reports exceed objective measurements obtained via actigraphy. For example, in the CARDIA study, the average subjective overestimation was approximately one hour greater than the objective actigraphy recording and adults with objective sleep durations of 5 h overestimated their sleep by 1.2 h whereas individuals objectively sleeping 7 h overestimated their sleep by 0.4 h [7]. However, despite the overall tendency to underestimate their sleep duration in this study, about half of participants who participated in screening for this study did not have objectively recorded sleep duration < 7 h. Therefore, these data demonstrate the importance of utilizing objective sleep duration screening prior to enrolling participants in sleep extension studies because participants often do not accurately self-identify their objective sleep duration, even when screened with several self-report questionnaires. The Bland–Altman analysis reinforced these results, showing modestly average underestimation but increasingly negative discrepancies with longer objective sleep durations, alongside a banding pattern consistent with rounded self-reports. Cubic spline modeling indicated that misperception follows a U-shaped pattern, with the lowest predicted probability of high subjective–objective difference at approximately 6.3 h of actigraphically recorded sleep.

In terms of predictors of sleep duration difference, participants with poorer objective sleep (longer sleep onset latency and higher wake after sleep onset), higher sleep disturbance, and higher perceived stress had more of a tendency to subjectively underestimate their sleep duration, which is consistent with previous studies. Results also indicated that actigraphically measured SE had a positive relationship with sleep duration difference when controlling for other actigraphy variables, in that higher SE was associated with less subjective underestimation. Given that sleep is by nature a state of reversible perceptual disengagement [24], it may be that individuals with poorer quality are more attuned to awakenings, even among this sample which was screened for insomnia symptoms. Although the average ISI score in this sample was low, the range extended to the subthreshold and mild insomnia ranges. It is possible that longer SOL and WASO may reflect a greater tendency to estimate sleep as wakefulness due to cognitive bias, cognitive, or physiologic hyperarousal [8, 9, 25]. Among studies of older adults, those with poorer cognitive function tend to overestimate their sleep duration and quality, perhaps because they do not recall their time awake at night. Therefore, having greater attention toward awakenings may lead to estimating more time awake in the night.

In comparing this study to previous research, it is important to consider the specific population (adults who applied to participate in a sleep intervention trial) and how both self-report and objective sleep duration were assessed. In contrast to epidemiologic studies, this sample included only individuals with self-reported sleep duration ≤ 7 h who were free from significant sleep apnea symptoms and did not have moderate or severe insomnia symptoms. On average, participants were in good health with mildly elevated blood pressure and did not have elevated sleep disturbance or sleepiness scores. In addition, studies have shown the type of measurement of self-report and objective measure impacts the correlation between these variables. Habitual sleep duration has been measured as a self-report of the past 30 days (e.g., habitual bedtime/wake time, sleep latency, wake after sleep onset) or on a prospectively completed sleep diary. Some previous studies demonstrated that sleep discrepancies are similar whether comparing a single retrospective item of habitual sleep duration (as used in this study) or diary-based sleep duration to objective assessments, usually actigraphy. On the other hand, Matthews (2018) [26] demonstrated retrospective reports of sleep duration had lower correlations with objective sleep (actigraphy or PSG) compared with prospective reports (i.e., sleep diary). Furthermore, retrospective reports about sleep duration were only moderately correlated (0.5). Similarly, St. Onge and colleagues [27] reported a stronger correlation between self-reported sleep and actigraphically derived TST when participants were asked specifics of bedtime, waketime etc. versus a single item. It is also important to consider that actigraphy itself is not the “ground truth” and on average is an overestimation of sleep duration compared to the gold standard polysomnography. When participants are still, actigraphy is likely to overestimate their sleep.

This study highlights the complexity of sleep perceptions versus objective sleep. For individuals who perceive their sleep duration is insufficient but objective sleep duration is adequate, their concerns may lead them to engage in problematic behaviors, such as taking hypnotics or spending excessive time in bed leading to worse sleep quality. In addition, others may perceive their sleep as adequate but not be aware of a need to improve their sleep, resulting in chronic lack of sleep and associated poor health outcomes [28, 29]. In studies of individuals with habitual objective short sleep duration who do not perceive dysfunction, they are indeed sleepy and when given the opportunity, they are able to extend their sleep and demonstrate improved performance [30, 31]. Therefore, more research is needed at the intersection between subjective and objective sleep, taking into account daytime sleepiness and perceived dysfunction, to help elucidate who is most at risk for the effects of habitual short sleep duration and direct target interventions.

An important limitation to consider in this study is that self-reported habitual sleep duration was assessed at prescreening, and then actigraphy was conducted at enrollment, which was usually one or more weeks later. In addition, it is possible that participants in this study may have changed their sleep behavior due to monitoring (Hawthorne effect) or under-reported their sleep to be eligible to participate in a study aimed at improving sleep. It is also important to consider that many participants in this study had sub-optimal sleep (< 7 h) but the average actigraphy derived sleep duration of 6.8 h was higher than reported in other epidemiologic studies. Furthermore, sleepiness and sleep-related impairment were in the non-impaired range, and results may have been different in a sample with shorter sleep or more impairment. This may be a reason that sleepiness was not associated with subjective–objective sleep duration difference. In addition, it is important to note that many analyses relate the difference between subjective and objective sleep duration to objective sleep duration or to measures correlated with objective sleep duration. As such, while these models accurately describe empirical associations between sleep difference and objective sleep characteristics, part of these relationships may reflect regression to the mean inherent in difference-score analyses rather than purely biological mechanisms. Accordingly, these findings should be interpreted as descriptive of sleep perception patterns rather than causal effects of objective sleep duration on misperception. Strengths of this study include collection of a large sample of adults with self-reported, short sleep duration as well as detailed psychosocial questionnaires. Although use of a single self-reported sleep duration item is a limitation, it also has clinical validity because this is how sleep is likely being assessed during medical visits.

## Conclusion

Results of this study show that on average, participants applying to participate in a sleep extension study underestimated their sleep compared to actigraphically measured sleep duration. About half of those who applied for the study slept more than 7 h when monitored with actigraphy. Participants with poorer subjective and objective sleep quality showed greater bias in underestimating their sleep, although this relationship was non-linear. Based on these findings, sleep extension interventions should include objective sleep assessments prior to enrollment, since self-reports are often inaccurate. Public health initiatives should also address sleep perception as a key factor in motivating individuals to change their sleep behaviors.

## Data Availability

Data are available upon written request to the corresponding author.

## Abbreviations

*STITCH*: Sleep Technology Intervention to Target Cardiometabolic Health
*PHQ-8*: Patient Health Questionnaire, 8-item
*ISI*: Insomnia Severity Index
*ESS*: Epworth Sleepiness Scale
*AHI*: Apnea-hypopnea index
*SOL*: Sleep onset latency
*WASO*: Wake after sleep onset
*SE*: Sleep efficiency
